# Plant Peptides on the Rise: From Historical Insight to Future Applications

**DOI:** 10.1111/pbi.70613

**Published:** 2026-02-23

**Authors:** Shunxi Wang, Jinghua Zhang, Minghui Chen, Bokai Zhang, Huina Zhao, Liuji Wu

**Affiliations:** ^1^ State Key Laboratory of High‐Efficiency Production of Wheat‐Maize Double Cropping, College of Agronomy Henan Agricultural University Zhengzhou China

**Keywords:** biomedicine, crop improvement, development, non‐canonical peptides, plant peptides, stress response

## Abstract

Plant peptides constitute a rapidly expanding class of signalling molecules essential to plant physiology, mediating key processes such as development, stress adaptation, and immune responses. This review traces the history of plant peptide research, from the seminal discovery of systemin to the recent identification of non‐canonical peptides (NCPs) translated from small open reading frames (sORFs) in non‐coding RNAs. We delineate the distinct biosynthetic pathways of canonical peptides (CPs), which undergo proteolytic processing and post‐translational modifications, and NCPs, which are directly translated, often without further processing. The diverse biological functions of these peptides span development, reproduction, abiotic stress tolerance, biotic defence, and antimicrobial activity. Furthermore, we discuss emerging agricultural applications, including genetic engineering of peptides, exogenous peptide application, and trait optimization informed by natural peptide variation. Beyond agriculture, many plant peptides exhibit therapeutic potential due to their antimicrobial and anticancer properties. Despite significant advances, challenges remain in functional validation, field application, and scalable production. Future progress will depend on the integration of multi‐omics approaches, artificial intelligence (AI)‐driven prediction, and precision genome editing to fully harness the transformative potential of plant peptides for crop improvement and novel biopharmaceuticals.

## 1 Introduction

Peptides, typically consisting of 2–100 amino acid residues, represent a rapidly expanding and functionally diverse class of plant signalling molecules (Tavormina et al. 2015). They are integral to a wide range of physiological processes, including responses to biotic and abiotic stresses, as well as cellular differentiation and development (Datta et al. 2024; Xiao et al. 2025; Yan et al. 2025; Zhang, Han, et al. 2025). For example, Rapid Alkalinization Factor 34 (RALF34) facilitates pollen tube rupture and sperm release by competing with RALF4/19 for binding to the BUPS1/2–ANXUR1/2 receptor complex, thereby triggering a signalling cascade essential for fertilisation (Ge et al. 2017). In rice, OsPep3 enhances resistance to brown planthopper (BPH) infestation and to fungal and bacterial pathogens through interaction with Plant Elicitor Peptide (PEP) Receptors (PEPRs) (Shen et al. 2022). Additionally, some plant peptides possess notable antimicrobial activity against both plant and human pathogens (Huang, Araujo, et al. 2021; Tian et al. 2021; Chen et al. 2025), underscoring their potential for cross‐species therapeutic applications.

Plant peptides are primarily classified by origin into canonical peptides (CPs) and non‐canonical peptides (NCPs) (Chong et al. 2020; Wang, Tian, et al. 2020; Chen et al. 2023). CPs originate from canonical open reading frames (ORFs) and are translated as precursor proteins (Olsson et al. 2019). These precursors undergo proteolytic processing and frequently require post‐translational modifications (PTMs), such as tyrosine sulfation, proline hydroxylation, and hydroxyproline arabinosylation, to yield bioactive mature peptides (Matsubayashi 2014; Tavormina et al. 2015). These PTMs are critical for CP function; for example, tyrosine sulfation is essential for the biological activity of phytosulfokine (PSK) in plant development and immunity (Kaufmann and Sauter 2019; Li, Di, et al. 2024). In contrast, NCPs are translated directly from small ORFs (sORFs) located within untranslated regions (UTRs) of mRNAs or non‐coding RNAs, including long non‐coding RNAs (lncRNAs), primary microRNAs (pri‐miRNAs), and circular RNAs (circRNAs), typically without extensive post‐translational processing (Couso and Patraquim 2017; Chong et al. 2020; Wang, Tian, et al. 2020). Recent studies have provided compelling evidence for the involvement of NCPs in plant development and stress responses (Sharma et al. 2020; Chen et al. 2025; Yu et al. 2025). For example, miPEP858a regulates the expression of pri‐miR858a and plays a critical role in plant development and the phenylpropanoid biosynthetic pathway (Sharma et al. 2020), underscoring the emerging significance of these novel molecular regulators.

The progression of plant peptide research has been driven not only by technological advancements but also by an evolving understanding of plant biology. The field was initiated in 1991 with the discovery of systemin, through bioassay‐guided purification (Pearce et al. 1991). This landmark finding demonstrated that peptides can function as potent, mobile signalling molecules in plants. Since then, additional peptide families have been identified using various approaches, including forward genetics and bioinformatics (Fletcher et al. 1999; Cock and McCormick 2001; Butenko et al. 2003; Huffaker et al. 2006; Huffaker et al. 2011; Campbell and Turner 2017). However, these traditional methods face significant challenges in detecting peptides, particularly NCPs. Recent advances in next‐generation sequencing and mass spectrometry (MS) have enabled the development of omics‐based strategies for large‐scale peptide identification, with particular efficacy in identifying NCPs. One such approach, peptidogenomics, integrates MS data with customised databases to facilitate genome‐wide discovery of plant peptides (Wang, Tian, et al. 2020; Pei et al. 2022). These innovations have deepened our understanding of plant biology and opened new avenues for enhancing crop resilience and productivity.

Given the increasingly recognised roles of plant peptides in stress responses and development, this review provides a comprehensive overview of the history of plant peptide research, as well as the biosynthesis and processing of CPs and NCPs. We summarise the molecular function of plant peptides in regulating growth, development, reproduction, environmental adaptation, as well as their antimicrobial properties. Furthermore, we explore the potential applications of plant peptides in agriculture and biomedicine. Finally, we address current challenges in functional characterisation, practical application, and propose future directions for leveraging plant peptides to improve plant adaptability and agricultural outcomes. Collectively, these insights are vital for advancing strategies that enhance plant resilience, productivity, and translational applications in biomedicine.

## 2 The History of Plant Peptide Research

**FIGURE 1:**
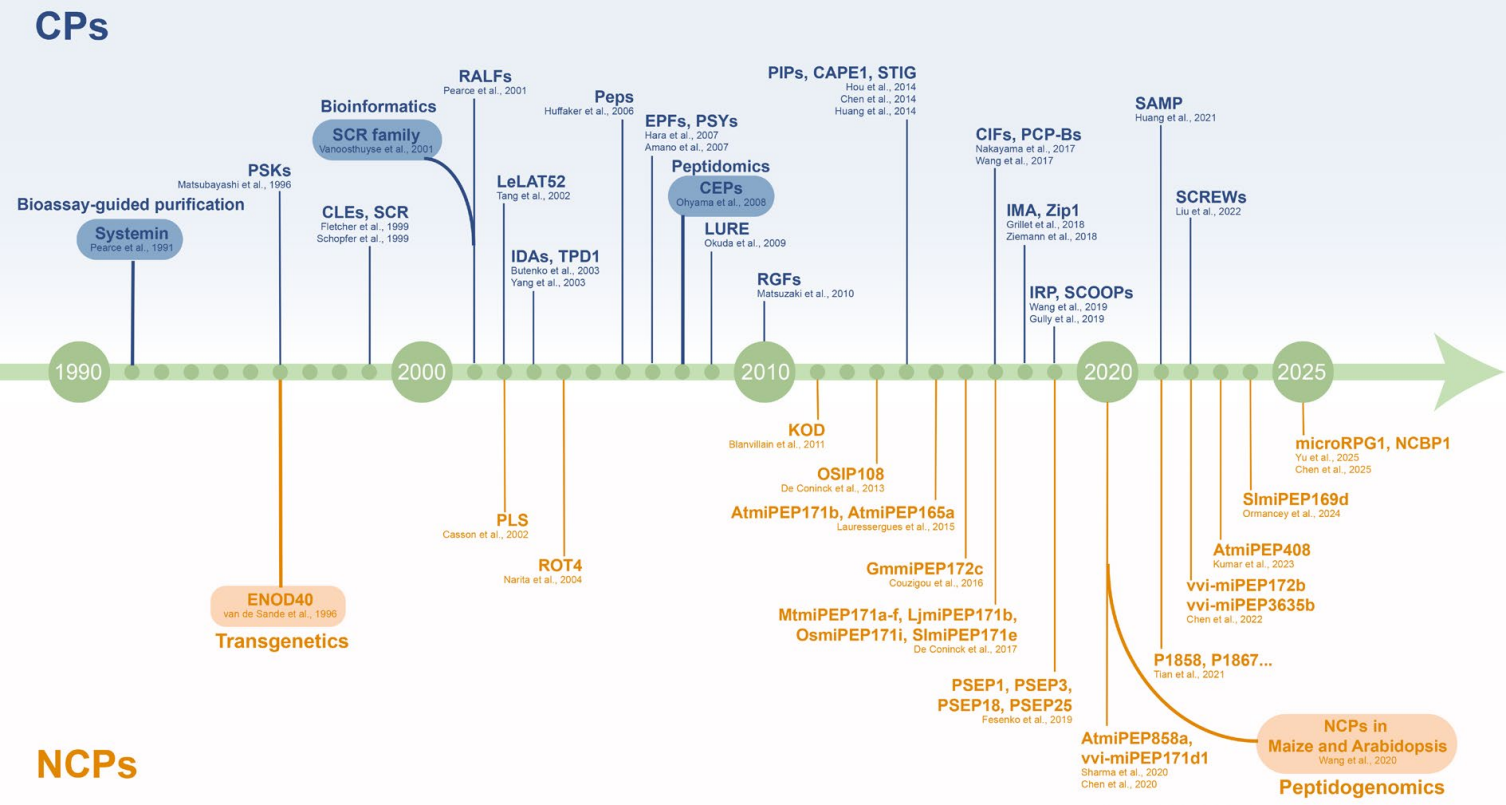
The history of canonical peptides (CPs) and non‐canonical peptides (NCPs) discovery in plants. The discovery of plant canonical peptides (CPs) began in 1991 with the identification of systemin. Early CPs were primarily uncovered through bioassay‐guided purification or genetic analyses. Subsequent advances in next‐generation sequencing and bioinformatics enabled the discovery of CP families, starting with S‐LOCUS CYSTEINE‐RICH PROTEIN LIKE (SCRL) and expanding to numerous others. By 2008, peptidomics emerged as a key omics methodology for plant CP characterisation. In parallel, non‐canonical peptide (NCP) research was initiated in 1996 with the identification of ENOD40. Subsequent studies uncovered additional NCPs in plants. Recently, advances in high‐throughput sequencing and mass spectrometry (MS) technologies, peptidogenomics was developed to large‐scale identification of NCPs in plants, significantly expanding our understanding of the plant peptidome.

Peptide research was inaugurated by the discovery of secretin in 1902. This 27‐amino acid peptide, discovered in dogs by Bayliss and Starling, was the first identified endogenous peptide (Bayliss and Starling 1902). A transformative milestone followed in 1921–1922 with the isolation of insulin by Banting and Best (Banting and Best 1922). However, plant peptide research began with the discovery of systemin in 1991 (Pearce et al. 1991), approximately 90 years after comparable research commenced in animals. The plant peptide research history reflects not only the advancement of analytical technologies but also a fundamental shift in our understanding of plant biology. This trajectory reveals a field that has matured alongside molecular biology and genomics, continually challenging long‐held assumptions about plant communication and regulatory complexity. In this section, we provide a historical overview of peptide signalling systems, highlighting key milestones that have shaped current knowledge (Figure 1).

### 2.1 The Molecular Biology Revolution: The Emergence of Canonical Peptides (CPs)

The field of plant peptide research was inaugurated in 1991 with the discovery of systemin, the first unequivocally identified plant peptide, isolated via bioassay‐guided purification from wounded tomato leaves. This 18‐amino acid peptide acted systemically to activate protease inhibitor genes, playing a key role in defence against herbivory (Pearce et al. 1991). The identification of systemin overturned the prevailing assumption that plant signalling was mediated exclusively by small molecules, establishing that plant peptides could function as potent, mobile signals. Subsequent research saw the emergence of other key peptide families. Phytosulfokines (PSKs), identified from *Asparagus* mesophyll cultures due to their mitogenic activity, were revealed to be sulfated pentapeptides that promote cell proliferation and differentiation (Matsubayashi and Sakagami 1996). Similarly, RALFs were discovered in tobacco based on their capacity to rapidly alkalinize culture media (Pearce et al. 2001). In parallel, forward genetic studies in *Arabidopsis thaliana* led to the identification of CLAVATA3 (CLV3), a secreted peptide that binds to receptor complexes to regulate shoot apical meristem size and stem cell homeostasis (Fletcher et al. 1999), highlighting the central role of peptides in plant development.

Beyond signalling peptides, plants also produce peptides with direct bioactivity, such as defensins and cyclotides. The term plant defensin was formally established in 1995, following advances in their structural and functional characterisation (Terras et al. 1995). Subsequent research in Arabidopsis identified key defensin genes (e.g., *PDF1.1*, *PDF1.2*, *PDF2.1*–*PDF2.3*), confirming them as a distinct and widespread peptide family (Penninckx et al. 1996; Epple et al. 1997; Thomma et al. 1998). Their most extensively documented function is broad‐spectrum antifungal activity against various plant pathogens, typically mediated through interactions with microbial membranes or intracellular targets (Thomma et al. 2002). In contrast, the history of cyclotides involves a significant gap between initial discovery and formal classification. Although the prototypic cyclotide, kalata B1, was first isolated from the African plant *Oldenlandia affinis* in the early 1970s (Gran 1973), the unifying term cyclotide was not introduced until 1999. This followed the recognition of their characteristic cyclic backbone as a widespread structural motif (Craik et al. 1999). Cyclotides primarily function as natural host‐defence agents in plants. Their dominant role is insecticidal activity, achieved through interactions with and disruption of cellular membranes, which interferes with normal biological processes in pests (Huang et al. 2019; Khatibi et al. 2025). Beyond this primary function, cyclotides exhibit a remarkably broad spectrum of bioactivities, including proteinase inhibition, cytotoxicity to tumour cells and antiviral effects (Pelegrini et al. 2007; Troeira Henriques et al. 2014). Taken together, defensins and cyclotides exemplify how plants have evolved diverse peptides to address distinct ecological threats, from fungal infections to herbivore predation.

### 2.2 The Omics and Bioinformatics Era: Expanding the Peptide Repertoire

The sequencing of the Arabidopsis genome in 2000 and subsequent plant genomes revolutionised peptide discovery. These genomic resources enabled large‐scale in silico mining for CPs, often based on features such as signal peptides, conserved motifs, and precursor protein structures (Lease and Walker 2010). This led to the identification of the S locus cysteine‐rich (SCR) peptides family in Arabidopsis (Vanoosthuyse et al. 2001). Other peptide families, including CLAVATA3/EMBRYO SURROUNDING REGIONs (CLEs) (Cock and McCormick 2001; Kucukoglu and Nilsson 2015; Li et al. 2019; Kang et al. 2022), PSKs (Lorbiecke and Sauter 2002; Shen, Stuhrwohldt, and Lin 2023; Li, Di, et al. 2024), Peps (Huffaker et al. 2006; Huffaker et al. 2011; Yamaguchi and Huffaker 2011), and RALFs (Bedinger et al. 2010; Campbell and Turner 2017; Liu et al. 2024), were found to possess numerous paralogs, suggesting extensive functional diversification and redundancy.

The advent of MS‐based peptidomics enabled direct, large‐scale identification of endogenous peptides (Clynen et al. 2001; Verhaert et al. 2001), and was first applied to identify novel peptides in plants in 2008 (Ohyama et al. 2008). A prime example of its subsequent application is a study in tomato, which identified dozens of defence peptides induced by wounding and methyl jasmonate treatment. Among them, a CAP‐derived peptide 1 (CAPE1), which is derived from the C‐terminal region of Pathogenesis‐Related protein 1b (PR1b), induces significant antipathogen and minor antiherbivore responses in tomato (Chen et al. 2014). Subsequent studies implicated CAPE1 in salt stress regulation (Chien et al. 2015). Most recently, we established a comprehensive and high‐confidence maize peptide atlas using peptidomics, providing novel insights into the dynamic expression patterns and tissue‐specific distribution of endogenous peptides (Ali et al. 2024). These technological innovations accelerated the pace of plant peptide discovery and functional characterisation.

### 2.3 The Emergence of Hidden Regulators: Non‐Canonical Peptides (NCPs)

For decades, sORFs within non‐coding RNAs were widely considered non‐functional or translational noise, largely excluded from canonical gene annotations. However, this view has undergone a profound transformation, catalysed by a series of paradigm‐shifting discoveries and methodological advances. The discovery of ENOD40 in 1996, encoded by a sORF within a lncRNA, demonstrated that functional peptides could arise from previously overlooked genomic regions. ENOD40 is critical for initiating symbiotic interactions with nitrogen‐fixing bacteria (van de Sande et al. 1996). Additional examples such as POLARIS (PLS) and ROTUNDIFOLIA4 (ROT4) further reinforced the functional relevance of NCPs in plant development and signalling (Casson et al. 2002; Narita et al. 2004).

The advent of ribosome profiling (Ribo‐seq) provided genome‐wide snapshots of actively translating sequences, independent of conventional gene annotations (Ingolia et al. 2009). These datasets revealed pervasive translation of sORFs across diverse plant genomes (Planchard et al. 2018; Wu et al. 2019; Kute et al. 2022). To systematically identify these previously hidden peptides, we developed an integrated peptidogenomics approach that combines MS‐based peptidomics with customised genomic databases. Applying this method, we identified thousands of NCPs in Arabidopsis and maize (Wang, Tian, et al. 2020), demonstrating their pervasive presence in plants. This approach was subsequently extended to other plants, further validating its broad applicability (Moyer et al. 2021; Pei et al. 2022; Han et al. 2024). To facilitate access and further research, we established a dedicated database, NCPBook, cataloguing NCPs from various species (Sami et al. 2024). These efforts have enabled large‐scale NCP discovery and reshaped our understanding of the coding potential within plant genomes.

## 3 Biosynthesis and Processing of Plant Peptides

Plant peptides are generally classified into CPs and NCPs, with each type undergoing distinct biosynthetic and processing pathways. Understanding how bioactive peptides are generated is essential for elucidating their biological roles. This section outlines the divergent biosynthesis and processing routes of CPs and NCPs.

### 3.1 Biosynthesis and Processing of Canonical Peptides (CPs)

**FIGURE 2:**
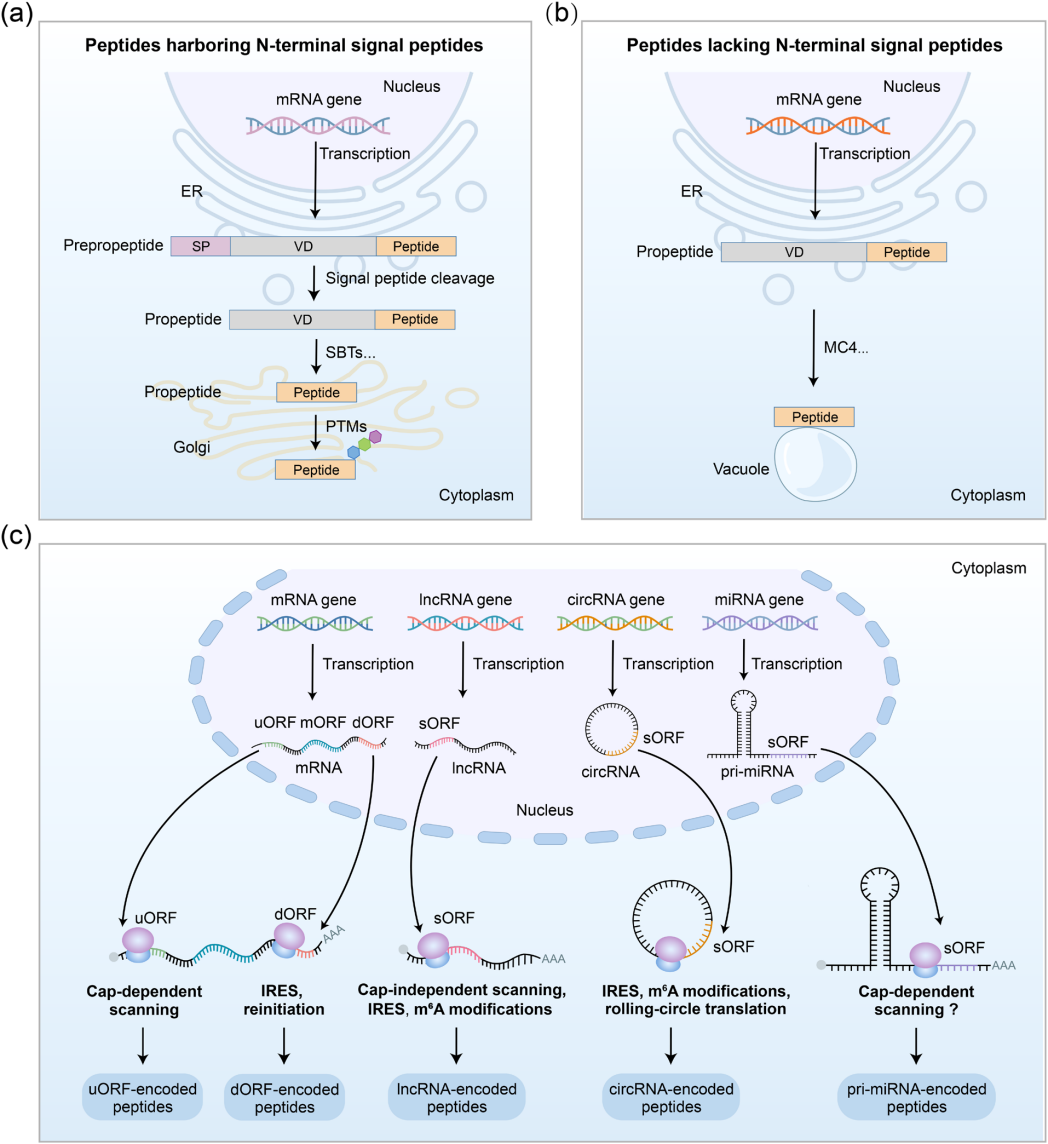
Biogenesis of canonical peptides (CPs) and non‐canonical peptides (NCPs) in plants. (a) Biogenesis and processing of plant CPs harbouring N‐terminal signal peptides. CPs harbouring N‐terminal signal peptides are translated as prepropeptides. These undergo signal peptide cleavage to form propeptides, which are subsequently processed by proteases such as subtilases (SBTs) and modified through post‐translational modifications (PTMs) to yield mature bioactive peptides. (b) Biogenesis and processing of plant CPs lacking N‐terminal signal peptides. The processing of CPs lacking N‐terminal signal peptides is less well characterised. A representative example is the biogenesis of Plant Elicitor Peptides (Peps), where propeptides are proteolytically processed by METACASPASE 4 (MC4) to produce mature peptides. (c) Biogenesis of plant NCPs. NCPs arise through direct translation of small open reading frames (sORFs) located in untranslated regions (UTRs) or within non‐coding RNAs, including long non‐coding RNAs (lncRNAs), circular RNAs (circRNAs), and primary microRNAs (pri‐miRNAs). This biogenesis occurs via specific translation mechanisms and operates independently of classical peptide maturation pathways.

CPs are initially transcribed from coding genes and translated into larger prepropeptides. These prepropeptides typically undergo a series of proteolytic processing and PTMs to yield mature, bioactive peptides (Matsubayashi 2014; Tabata and Sawa 2014; Stührwohldt, Ehinger, et al. 2020; Stintzi and Schaller 2022) (Figure 2a). All identified CPs require proteolytic cleavage to release the mature peptide. The processing mechanisms of CP propeptides remained unclear until 2008, when the PSK4 precursor was found to undergo N‐terminal cleavage by *Arabidopsis thaliana* subtilases 1.1 (*AtSBT1.1*) (Srivastava et al. 2008). Proteolytic enzymes involved in CP maturation include a variety of protease families, such as metacaspases, papain‐like enzymes, carboxypeptidases, subtilisin‐like proteases, and subtilases (SBTs) (Matsubayashi 2012; Schardon et al. 2016; Chen, Fan, et al. 2020; Stührwohldt, Scholl, et al. 2020; Liu et al. 2023; Deng et al. 2025). For example, AtSBT3.8 processes SERINE‐RICH ENDOGENOUS PEPTIDE 20 (SCOOP20) by cleaving the N‐terminal portion of the precursor, while AtSBT3.5 processes SCOOP12 similarly (Yang, Kim, et al. 2023). Additionally, the papain‐like cysteine protease TaRD21A processes a precursor to release the mature peptide Wip1, thereby conferring resistance to wheat yellow mosaic virus (Liu et al. 2023).

The processing of CPs varies depending on the presence or absence of N‐terminal signal peptides. CPs containing N‐terminal signal peptides are typically processed by proteases during secretion or within the apoplast (Stührwohldt, Scholl, et al. 2020). In contrast, the processing of CPs lacking N‐terminal signal peptides remains less well understood. A well‐characterised example is Pep1, which is cleaved from its precursor PROPEP1 by METACASPASE 4 (MC4) following a sustained cytosolic Ca^2+^ influx, triggering a defence response via receptor binding (Hander et al. 2019; Shen et al. 2019; Zhu, Yu, et al. 2020) (Figure 2b). Recent evidence further indicates that SUMOylation of PROPEP1 is critical for its proper processing (Zhang, Wu, et al. 2025). Similarly, in maize, the papain‐like cysteine proteases CP1 and CP2 are required to process and release the active mature peptide *Zea mays* immune signalling peptide 1 (Zip1) (Ziemann et al. 2018). Despite the identification of only a limited number of CPs lacking N‐terminal signal peptides, they are functionally important in plants, with their processing mechanisms still largely unclear.

In addition to proteolytic cleavage, CP maturation can involve PTMs such as tyrosine sulfation, proline hydroxylation, and hydroxyproline arabinosylation (Matsubayashi 2014; Royek et al. 2022). Tyrosine sulfation, catalysed by tyrosyl protein sulfotransferase (TPST), is essential for the bioactivity of certain peptides like PSKs (Matsubayashi and Sakagami 1996; Moore 2003). Proline hydroxylation, catalysed by prolyl‐4‐hydroxylase (P4H) (Myllyharju 2003), and subsequent arabinosylation of hydroxyproline residues are involved in the maturation of peptides like PLANT PEPTIDE CONTAINING SULFATED TYROSINE 1 (PSY1) (Amano et al. 2007) and CLV3 (Kondo et al. 2006). Additionally, correct disulfide bond formation is facilitated by protein disulfide isomerases (PDIs) (Gruber et al. 2006). However, whether PDIs contribute to disulfide bond formation in cysteine‐rich peptides remains unclear. Beyond these linear peptides, cyclotides are a unique class of head‐to‐tail cyclic peptides. Their biosynthesis in plants involves asparaginyl endopeptidases (AEPs), which catalyse backbone cyclization through transpeptidation (Khatibi et al. 2025; Zhou et al. 2025).

### 3.2 Biosynthesis and Processing of Non‐Canonical Peptides (NCPs)

The biosynthesis and processing of NCPs differ fundamentally from those of CPs. NCPs are defined as peptides translated directly from sORFs located in UTRs, lncRNAs, circRNAs, and pri‐miRNAs. Their discovery and characterization represent a paradigm shift in molecular biology. While utilizing diverse and often non‐canonical translation mechanisms, these functional NCPs constitute a hidden peptidome with significant biological roles (Figure 2c).

#### 3.2.1 UTR‐Encoded NCPs

Although eukaryotic mRNA UTRs were once viewed solely as untranslated regulators of translation, stability, and tissue‐specific expression (Hardy and Balcerowicz 2024), recent studies reveal that both 5′ UTRs and 3′ UTRs harbour translatable sORFs called upstream ORFs (uORFs) and downstream ORFs (dORFs), respectively (Zhang, Wu, et al. 2020; Liang et al. 2021; Guo, Chen, et al. 2023; Hermesch et al. 2024; Wu et al. 2024). Translation initiation in these regions is influenced by factors like the sequence context around the start codon, the ORF length, and its proximity to the main coding sequence. uORFs are often translated via the canonical cap‐dependent scanning mechanism (Hinnebusch et al. 2016; Zhang, Wang, and Lu 2019). Translation of dORFs in the 3′ UTR is less common but may involve mechanisms like internal ribosome entry sites (IRESs) or reinitiation (Dodbele and Wilusz 2020; Wu, Wright, et al. 2020). Translated sORFs within UTRs primarily function as *cis*‐ or *trans*‐regulatory elements. uORFs generally repress the translation of the main ORF (mORF) (von Arnim et al. 2014; Xu, Greene, et al. 2017; Zhang, Wu, et al. 2020; Wu et al. 2022; Tian et al. 2024), whereas dORFs are often associated with enhanced mORF translation (Wu, Wright, et al. 2020; Guo, Chen, et al. 2023; Wu et al. 2024). Engineered translatable ORFs (uORFs/dORFs) have proven effective in optimising crop performance, demonstrating the potential of translational control for enhancing agricultural yields (Xu, Uan, et al. 2017; Chen et al. 2023; Tian et al. 2024; Wang, Liu, and Guo 2024; Yang et al. 2024; Mou et al. 2025; Zhang, Xiang, et al. 2025). In humans, some uORF‐encoded peptides have been shown to function independently of their associated mORFs (Huang, Li, et al. 2021; Li, Yang, et al. 2024). However, the precise roles of UTR‐encoded peptides in plants remain largely uncharacterized and require further investigation.

#### 3.2.2 LncRNA‐Encoded NCPs

LncRNAs are operationally defined as transcripts exceeding 200 nucleotides (nt) with low protein‐coding potential. This size threshold historically facilitated their biochemical purification by enriching for polyadenylated RNAs and depleting infrastructural RNAs (Mattick et al. 2023). However, accumulating evidence demonstrates that many lncRNAs contain sORFs capable of encoding functional NCPs (Xu, Greene, et al. 2017; Wu et al. 2019; Guo, Chen, et al. 2023; Zhu et al. 2023). Translation of these sORFs occurs via cap‐independent mechanisms, notably IRES, which recruit ribosomes and initiate assembly without a 5′ cap (King et al. 2010; Wen et al. 2024). Additionally, endogenous *N* ^6^‐methyladenosine (m^6^A) modifications on lncRNAs drive widespread translation, experimentally validated through m^6^A site mutagenesis that ablates peptide production (Wu, Mo, et al. 2020; Pei et al. 2023). Supported by advances in ribo‐seq, computational prediction, and MS, functional lncRNA‐encoded peptides have now been identified across diverse plant species (Fesenko et al. 2019; Wu et al. 2019; Sruthi et al. 2022; Zhu et al. 2023; Wu et al. 2024; Li et al. 2025). For example, lncRNA‐derived peptides regulate cell fate specification and development in *Physcomitrella patens* (Fesenko et al. 2019). These findings significantly expand the known functional repertoire of lncRNAs and reveal a hidden proteome within eukaryotic transcriptomes.

#### 3.2.3 CircRNA‐Encoded NCPs

CircRNAs are a class of non‐coding RNAs generated through atypical reverse splicing (Qu et al. 2017; Zhao et al. 2019). First identified in viruses, circRNAs have since been recognized for their diverse regulatory roles, such as acting as miRNA sponges that modulate the miRNA–mRNA axis and functioning as transcriptional regulators (Kolakofsky 1976; Jeck and Sharpless 2014; Lv et al. 2020). Due to their covalently closed loop structure, lacking both a 5′ cap and a 3′ poly(A) tail, circRNAs are resistant to exonuclease degradation and were long thought to lack translational potential (Patop et al. 2019). However, recent studies have revealed that circRNAs harbor sORFs capable of encoding peptides (Wu, Mo, et al. 2020; Wen et al. 2024; Yi et al. 2024; Pan et al. 2025). Translation of these sORFs can occur via IRES, m^6^A modifications, or through a unique endogenous rolling‐circle translation mechanism, which terminates at codons located outside the circular structure (Gao et al. 2021; Liu, Li, et al. 2021). These mechanisms underscore the complex and multifaceted biological functions of circRNAs. While circRNA‐encoded peptides have been extensively characterized in mammals (Wu, Mo, et al. 2020; Huang, Zhu, et al. 2024; Huang, Li, et al. 2024; Yi et al. 2024), their roles in plants remain largely unexplored. To date, WRKY9‐88aa, a peptide encoded by a circRNA in rice, represents the only documented example, playing a key role in broad‐spectrum disease resistance (Pan et al. 2025). Further identification and functional characterization of plant circRNA‐encoded peptides are needed.

#### 3.2.4 Pri‐miRNA‐Encoded NCPs

While mature miRNAs are non‐coding, pri‐miRNAs, as precursors to mature miRNAs, possess characteristic features similar to mRNAs, including a hairpin structure, a 5′ cap, and a 3′ poly(A) tail (Achkar et al. 2016). Recent findings have revealed that pri‐miRNAs also contain sORFs capable of encoding NCPs, which play important functional roles (Couzigou et al. 2016; Chen, Deng, et al. 2020; Prasad et al. 2021; Ren et al. 2021; Chen et al. 2022; Lauressergues et al. 2022; Gautam et al. 2023; Kumar et al. 2023; Ormancey et al. 2024). Translation on these precursors is likely cap‐dependent for pri‐miRNAs, although this process is less well understood due to the presence of stem‐loop structures that hinder ribosome scanning (Yadav et al. 2021). Pri‐miRNA‐encoded NCPs function primarily by boosting transcription of their own miRNA genes, thereby reinforcing miRNA‐mediated regulatory loops (Gautam et al. 2023). Since the first discovery of pri‐miRNA‐encoded NCPs in 2015, numerous such peptides have been identified in plants (Lauressergues et al. 2015; Ormancey et al. 2021; Yadav et al. 2021). For instance, miPEP858a, encoded by pri‐miR858a, upregulates pri‐miR858a expression and plays a key role in regulating plant development and the phenylpropanoid biosynthetic pathway (Sharma et al. 2020). Another example is that treatment with synthetic miPEP172c increased nodule number in soybean without affecting other root development features (Couzigou et al. 2016), highlighting the importance of pri‐miRNA‐encoded NCPs in improving agronomic traits of crop plants.

Unlike CPs, which typically require proteolytic cleavage from larger precursors to attain functionality, NCPs often function as bioactive entities immediately following translation (Chen et al. 2023; Xiao et al. 2025). Furthermore, their characteristically shorter length means most NCPs lack classical N‐terminal signal peptides, precluding endoplasmic reticulum (ER) translocation and secretion via the conventional pathway. This suggests NCPs either function intracellularly or utilize unconventional secretory mechanisms (Hu et al. 2021). Despite their small size, NCPs may require proteolytic processing and can carry PTMs, although direct evidence for these processing events in NCPs is currently limited and represents a key gap in understanding their biosynthesis.

## 4 Biological Function of Plant Peptides

Since the discovery of the first plant signalling peptide, systemin, in 1991 (Pearce et al. 1991), extensive research has highlighted the critical roles of small peptides in plant growth, reproduction, stress response, and antimicrobial activity (Xiao et al. 2025; Zhang, Han, et al. 2025). This section reviews the major peptide families and their functions in different biological contexts, including both CPs (Table 1) and emerging NCPs (Table 2).

**TABLE 1:** Representative families of canonical peptides (CPs) with known biological roles in plants.

Peptide	Key receptor	Function	References
RGFs/GLVs	RGFR1/RGFR2/RGFR3/RGI1‐5	RAM maintenance; lateral root development; root gravitropism; root elongation and cell division; root hair growth; immune response	(Matsuzaki et al. 2010; Stegmann et al. 2022; Xu et al. 2023)
PSKs	PSKR1/PSKR2	Cells proliferation; root elongation; lateral root initiation; nodulation; pollen germination and tube growth; fruit ripening and fruit quality; drought‐induced flower drop; calcium influx; immunity; heat stress; rhizobia proliferation; drought stress	(Yamakawa et al. 1999; Kutschmar et al. 2009; Zhang, Han, et al. 2025)
CLEs	ACR4/CIKs/CLV1/CLV2/BAMs/TDR	Columella stem cells differentiation; stem cell maintenance; protophloem development; root growth; lateral root primordium development; lateral root growth and elongation; SAM size and fruit development; seed production; dehydration resistance; cambial cell division and xylem differentiation; root growth and protophloem differentiation; pathogenic bacteria resistance; root nodules; root architecture; pollen tube growth	(Stahl et al. 2009; Gutiérrez‐Alanís et al. 2017; Kim, Jeon, and Kim 2021; Kwon et al. 2022)
PSYs	PSYR1/PSYR2/PSYR3	Root elongation; root growth; immune response; cell elongation	(Amano et al. 2007; Ogawa‐Ohnishi et al. 2022; Yimer et al. 2023)
CEPs	CEPR1/CEPR2/RLK7/CRA2	Lateral root growth and development; drought tolerance; nitrate uptake under; root meristem activity; primary root development; Ca^2+^ signalling; MAPK signalling	(Delay et al. 2013; Tabata et al. 2014; Luo et al. 2023)
RALFs	FER/ANJ/BUPS1/2/ANX1/2/FER/CVY1/ANJ/HERK1	Calcium‐dependent signalling; root growth; pollen tube growth; rupture; hybridization; intergeneric hybridization; salt tolerance; immunity; ROS generation; pollen hydration; preventing undesired pollen tube penetration; outcompeting stigmatic RALFs and enabling successful pollen tube penetration; pollen tube rupture and sperms release; lateral root growth; cell wall homoeostasis; media alkalinization; antagonises PAMP‐induced ROS; disease resistance	(Ge et al. 2017; Stegmann et al. 2017; Gjetting et al. 2020; Chao et al. 2023; Lan et al. 2023)
EPFs	ER/ERL1/ERL2	Stomatal development; ovule spacing; inflorescence and stem growth; cotyledon development; fruit development; meristemoid differentiation; stamen filament elongation; stamen elongation; self‐pollination and reproductive	(Hara et al. 2007; Hara et al. 2009; Xiong et al. 2022; Negoro et al. 2023)
CIFs	SGN3/GSO1/GSO2	Pollen wall formation; embryonic cuticle formation; lignification for Casparian strip development; polarises tapetal cells for sporopollenin; deposition in pollen wall formation; maintains embryonic cuticle integrity for seed protection	(Tsuwamoto et al. 2008; Truskina et al. 2022; Zhang, Han, et al. 2025)
IDAs/IDLs	SERK1/SERK2/BAK1/SERK3/SERK4/HSL2/HAE/HAE‐like	Disease resistance; lateral root emergence; drought tolerance; oxidative stress; floral organ abscission; root cap sloughing; cell separation; detachment of floral organs, fruits and leaves; root elongation; immunity	(Butenko et al. 2003; Vie et al. 2015; Lalun et al. 2024)
LURE	MDIS1/MIK1/2/LIP1/2/PRK3/6	Pollen tube growth; species‐specific male–female communication; precise navigation and successful fertilisation	(Okuda et al. 2009; Takeuchi and Higashiyama 2016; Yang et al. 2022)
CAPE1	Unknown	Salt tolerance; insect resistance; immunity; disease resistance	(Chen et al. 2014; Chien et al. 2015; Sung et al. 2021)
PIPs	RLK7	Salt tolerance; immunity; disease resistance	(Hou et al. 2014; Zhou et al. 2022; Yang, Wang, et al. 2023)
SCOOPs	MIK2‐BAK1/SERK4	Disease resistance; immunity	(Gully et al. 2019; Hou, Liu, Huang, et al. 2021)
Systemins	SYR1	Defence against pests; disease resistance; media alkalinization	(Pearce et al. 1991; Aprile et al. 2022)
PEPs	PEPR1/PEPR2	Immunity; extracellular alkaline conditions; salt tolerance	(Huffaker et al. 2006; Huffaker et al. 2011)
Zip1	unknown	Immunity; disease resistance	(Ziemann et al. 2018)
SCREWs	NUT	Immunity; stomatal aperture; drought stress	(Liu, Hou, et al. 2022)
SAMP	unknown	Disease resistance; immunity	(Huang, Araujo, et al. 2021)
IMA	Unknown	Fe homeostasis; Cd tolerance; Cu homeostasis; symbiotic nitrogen fixation	(Grillet et al. 2018; Ito et al. 2024)
TPD1	EMS1	Cell differentiation; tapetum development; female gametophyte development	(Chen et al. 2019)
PCP‐Bs	FER/ANJ	Repressing ROS production; pollen hydration	(Liu, Shen, et al. 2021)
SCR/SP11	SRK	Self‐incompatibility response	(Takayama and Isogai 2005)
STIG	PRK1/2	Regulating pollen tube growth	(Huang et al. 2014)
LeLAT52	LePRK2	Pollen hydration and germination	(Tang et al. 2002)
IRP	Unknown	Immunity	(Wang, Yao, et al. 2020)

**TABLE 2:** Non‐canonical peptides (NCPs) with experimentally validated biological functions in plants.

Peptide	Sequence	Function	References
ENOD40	MELCWLTTIHGS/MVLEEAWRERGVRGEGAHSSHSLT	Control sucrose Utilisation in nitrogen‐fixing nodules	(van de Sande et al. 1996; Rohrig et al. 2002)
PLS	MKPRLCFNFRRRSISPCYISISYLLVAKLFKLFLIH	Leaf vascular patterning	(Casson et al. 2002)
ROT4	MAPEENGTCEPCKTFGQKCSHVVKKQRAKFYILRRCIAMLVCWHDQNHDRKDS	Reduces cell proliferation and alters leaf shape	(Narita et al. 2004)
KOD	MWWLVGLTPVELIHLCTFRERLCHL	Reduced programmed cell death; affects both vegetative and reproductive growth	(Blanvillain et al. 2011)
PSEP1	MVQPLLARLASAAEFVALPGAILVAYFSTSRSTEPKRDHRK	Growth and development	(Fesenko et al. 2019)
PSEP3	MVHQDNSGSGLRSFNHPNPPPNNNRPPSNPPVVRNPSSGRTPHPYPPPPHNYNGYPN	Growth and development	(Fesenko et al. 2019)
PSEP18	MQAFTDTQGYSSFNGPATTAATTPPEVVGEFGGKGWRPSS	Growth and development	(Fesenko et al. 2019)
PSEP25	MVQSKQGLSLLKFIPKVIRPQTSDVSSAVLWGTTAACGALWLVQPFDWIKEQITGPKEESK	Growth and development	(Fesenko et al. 2019)
microRPG1	MILLPNHSRAPTNKRAGLQNYCPQRSCIRRG	Kernel dehydration	(Yu et al. 2025)
OSIP108	MLCVLQGLRE	Oxidative stress tolerance	(De Coninck et al. 2013)
AtmiPEP171b	MLLHRLSKFCKIERDIVYIS	Root development	(Lauressergues et al. 2015)
AtmiPEP165a	MRVKLFQLRGMLSGSRIL	Root development	(Lauressergues et al. 2015)
WRKY9‐88aa	MGANGGSTGRKWORGIHARERTTDAOWLSDAOSGSRHTGPRRRRRRRRSNSWSRERRPRRRPPPTRTWORPTTRPAAAAATATRRPLR	Enhances resistance to blast disease and bacterial leaf blight	(Pan et al. 2025)
AtmiPEP858a	MGGIESLLFTIVRDIGRYGTVCVVYNIKCVYTTRTKASTRTSHP	Flavonoid biosynthesis and development	(Sharma et al. 2020)
AtmiPEP408	MYFGSYHVAAKLFLSTFRFNTHSRKNQNPPANLEG	Stress resistance	(Kumar et al. 2023)
vvi‐miPEP171d1	MGYGTTP	Root formation	(Chen, Deng, et al. 2020)
vvi‐miPEP172b	MTSSSLSRQTKPYTSH	Cold resistance	(Chen et al. 2022)
vvi‐miPEP3635b	MFLYFIFRQLV	Cold resistance	(Chen et al. 2022)
NCBP1	KPWLRVALCPG	Antibacterial and disease resistance	(Chen et al. 2025)
P1858	KQRKKILGCTY	Antifungal activity	(Tian et al. 2021)
P1867	KAMGKLKVVLL	Antifungal activity	(Tian et al. 2021)
P52	KPWLRVALCPG	Antifungal activity	(Tian et al. 2021)
P1573	ITACVHLPA	Antifungal activity	(Tian et al. 2021)
P280	KSWRPSAMWSLKSLSA	Antifungal activity	(Tian et al. 2021)
P419	KCHLHPLCKGEALELFSVG	Antifungal activity	(Tian et al. 2021)
P1695	AGPPRGGPRARGGHGGR	Antifungal activity	(Tian et al. 2021)
P499	KVGFLPLHGMIQM	Antifungal activity	(Tian et al. 2021)
P1060	KPCYNLLGHKY	Antifungal activity	(Tian et al. 2021)
P423	AEAAGAKVV	Antifungal activity	(Tian et al. 2021)
P448	NKSSSKATPPHSRC	Antifungal activity	(Tian et al. 2021)
P509	IRTRGRPQ	Antifungal activity	(Tian et al. 2021)
P619	VLVDLFAREGH	Antifungal activity	(Tian et al. 2021)
P638	QIGVLLSAAPKL	Antifungal activity	(Tian et al. 2021)
P747	AKTRGDLIPP	Antifungal activity	(Tian et al. 2021)
P919	NAVREPSAG	Antifungal activity	(Tian et al. 2021)
P983	KLSLNWIGIR	Antifungal activity	(Tian et al. 2021)
P1063	QSKNFLPSTSEALQKS	Antifungal activity	(Tian et al. 2021)
P1240	KKGAAETKLSEE	Antifungal activity	(Tian et al. 2021)
P1215	EPIILGC	Antifungal activity	(Tian et al. 2021)
P1268	KVSIHYV	Antifungal activity	(Tian et al. 2021)
P1732	KALPHTPKGFF	Antifungal activity	(Tian et al. 2021)
P1716	DDPTVITNPLT	Antifungal activity	(Tian et al. 2021)
P1916	KPSMTKQLTSTHAFAVS	Antifungal activity	(Tian et al. 2021)
P1961	KDLLDKIP	Antifungal activity	(Tian et al. 2021)
GmmiPEP172c	MWVLCLFCWPTYTHGS	Increase nodule number	(Couzigou et al. 2016)
LjmiPEP171b	MYHRSKAKLCQTDGDDGGGSDM	Increase mycorrhization rate	(Couzigou et al. 2017)
MtmiPEP171a	MKKFEFPSAF	Decrease mycorrhization rate	(Couzigou et al. 2017)
MtmiPEP171b	MLLHRLSKFCKIERDIVYIS	Increase mycorrhization rate	(Couzigou et al. 2017)
MtmiPEP171c	MVNLYFV	Decrease mycorrhization rate	(Couzigou et al. 2017)
MtmiPEP171d	MHMYLK	Decrease mycorrhization rate	(Couzigou et al. 2017)
MtmiPEP171e	MMVFGKPKKAMLVRFNPKTDLHV	Decrease mycorrhization rate	(Couzigou et al. 2017)
MtmiPEP171f	MSHID	Decrease mycorrhization rate	(Couzigou et al. 2017)
OsmiPEP171i	MIARYIEREMTSKLGRGRKRAARLVAVFLLG	Increase mycorrhization rate	(Couzigou et al. 2017)
SlmiPEP171e	MKLGNIEGTYFIICLGRYI	Increase mycorrhization rate	(Couzigou et al. 2017)
SlmiPEP169d	MEGNGTLSFA	Immunity and disease resistance	(Ormancey et al. 2024)

### 4.1 Regulation of Growth and Development

**FIGURE 3:**
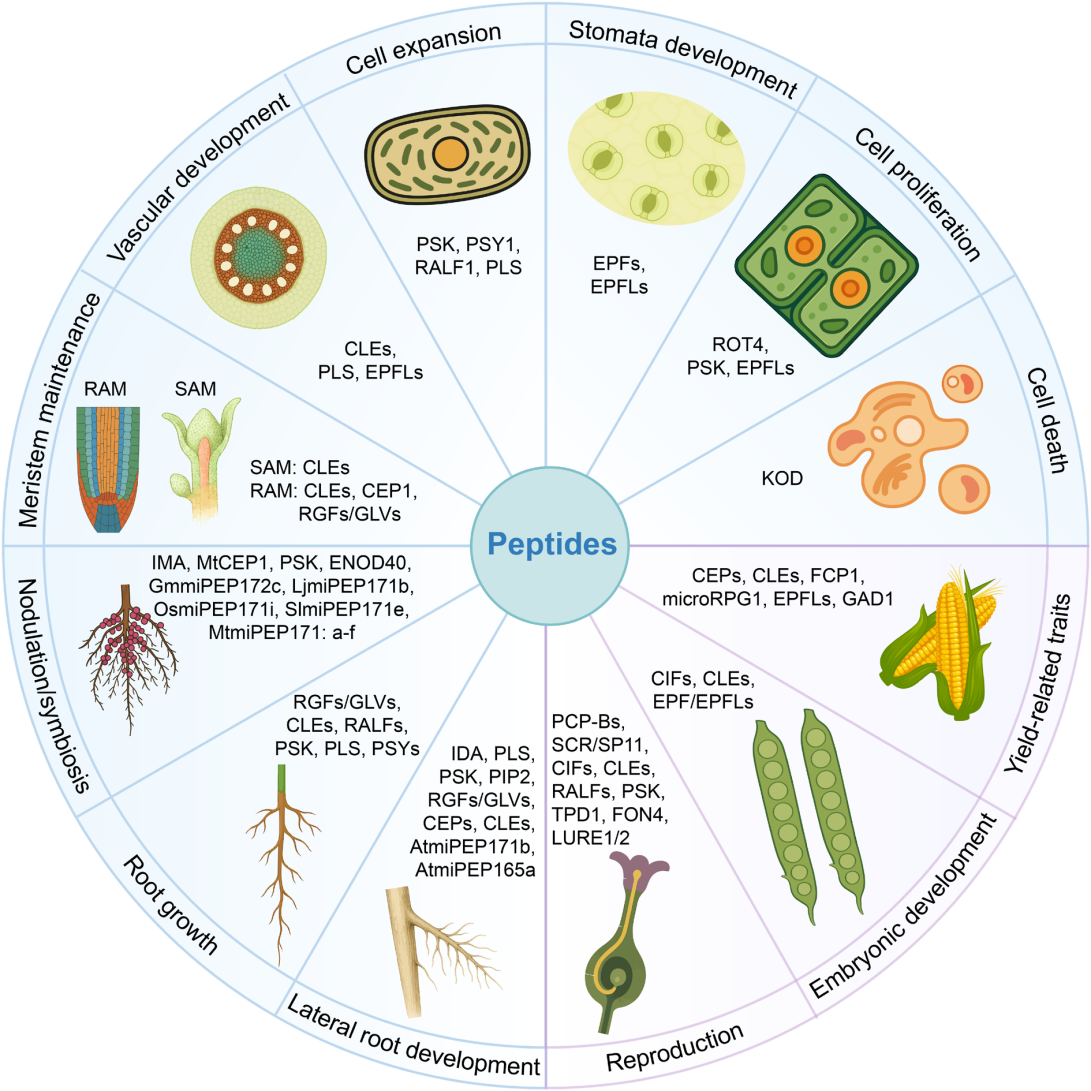
Plant peptides regulate development and reproduction processes. The schematic diagram categorises representative plant peptides based on their roles in key developmental and reproductive processes. Highlighted functions include vascular development, shoot/root apical meristem regulation, cell expansion, cell proliferation, cell death, embryonic development, and yield‐related traits. The recurring appearance of specific peptides, such as PSK, CLEs, RGFs/GLVs, RALFs, EPFLs, across multiple categories underscores their functional versatility in coordinating plant growth.

Plant development is orchestrated through the integration of internal and external signals, with peptides acting as essential mobile messengers (Figure 3). A prominent example is the CLE peptide family, which maintains stem cell homeostasis in shoot, root, and vascular meristems (DeYoung et al. 2006; Kinoshita et al. 2010; Nimchuk et al. 2015; Anne et al. 2018; Gregory et al. 2018; Hu, Zhu, et al. 2018; Ren et al. 2019; Schlegel et al. 2021; Song et al. 2021; Dao et al. 2022). In rice, the CLE family member FLORAL ORGAN NUMBER 4 (FON4), orthologous to Arabidopsis CLV3, maintains meristem determinacy and spikelet fertility, with mutations causing excess floral organs (Xu, Tao, et al. 2017). C‐TERMINALLY ENCODED PEPTIDEs (CEPs) promote primary and lateral root development across species (Roberts et al. 2013; Roberts et al. 2016; Taleski et al. 2018; Chapman et al. 2019; Huang et al. 2023; Selby and Jones 2023; Taleski et al. 2024). In rice, OsCEP6.1 negatively regulates tiller number, plant height, and grain weight, reinforcing the role of CEP peptides in balancing vegetative and reproductive growth (Sui et al. 2016). ROOT MERISTEM GROWTH FACTOR (RGF)/GOLVEN (GLV) peptides maintain the root meristem by activating PLETHORA transcription factors (Fernandez et al. 2013; Ou et al. 2016; Fernandez et al. 2020; Fang et al. 2021; Jourquin et al. 2022; Jourquin et al. 2023), while RALF peptides regulate root growth, alkalinization, and other developmental pathways (Pearce et al. 2001; Srivastava et al. 2009; Bergonci et al. 2014; Du et al. 2016; Zhu, Estévez, et al. 2020; Abarca et al. 2021; Li, Chen, et al. 2022). For example, GhRALF1, which is expressed during early fibre development, suppresses elongation by antagonising cell wall loosening in cotton (Wang, Hu, et al. 2023).

IDA and IDA‐LIKE (IDL) peptides mediate abscission by activating HAESA (HAE)/HAESA‐LIKE 2 (HSL2) receptor kinases (Patharkar and Walker 2015, 2016; Santiago et al. 2016; Li, Shi, et al. 2021; Wang, Wu, et al. 2023). Meanwhile, CASPARIAN STRIP INTEGRITY FACTOR (CIF) peptides are crucial for the integrity of the Casparian strip and related structures such as the embryonic cuticle and pollen wall (Doblas et al. 2017; Nakayama et al. 2017; Zhang et al. 2024). In contrast, the EPIDERMAL PATTERNING FACTOR (EPF)/EPF‐LIKE (EPFL)/STOMAGEN family primarily regulates stomatal patterning, vascular differentiation, and organ elongation (Hara et al. 2007; Hara et al. 2009; Hunt et al. 2010; Lee et al. 2015; Fischer and Teichmann 2017; Ikematsu et al. 2017; Zeng et al. 2020). In rice, the GRAIN NUMBER, GRAIN LENGTH AND AWN DEVELOPMENT1 (GAD1) peptide, homologous to Arabidopsis EPFLs, plays a role in controlling multiple yield‐related traits. It regulates grain number per panicle, grain length, and awn development (Jin et al. 2016; Jin et al. 2023). Furthermore, PSK and PSY peptides, post‐translationally modified and recognized by PSKR and PSYR receptors, regulate cell division and root growth (Matsubayashi et al. 2002; Amano et al. 2007; Stührwohldt et al. 2015; Hao et al. 2023; Shen, Stuhrwohldt, and Lin 2023).

Recent discoveries also emphasise the developmental roles of NCPs, including those encoded by pri‐miRNAs and lncRNAs. For instance, miPEP165a, derived from the pri‐miR165a, has been shown to promote primary root elongation in Arabidopsis by enhancing the transcription of its corresponding miRNA, establishing a positive feedback loop in root development (Lauressergues et al. 2015). Similarly, in grapevine ( *Vitis vinifera* ), miPEP171d1 facilitates adventitious root formation by activating GRAS‐family transcription factors involved in root initiation and development (Chen, Deng, et al. 2020). These findings suggest that miPEPs function as upstream regulators of transcriptional networks, linking peptide signalling to small RNA pathways. Beyond miPEPs, lncRNA‐derived peptides also play key roles in plant organogenesis and symbiotic interactions. The ENOD40 gene, initially characterised in legumes, encodes small peptides that modulate nodulation by interacting with RNA‐binding proteins and influencing cellular localization and signalling in response to nitrogen availability (Rohrig et al. 2002). These findings expand the landscape of peptide‐mediated developmental control in plants.

Recent studies have revealed that certain small peptides regulate plant development through interactions with plant hormones, including auxin, abscisic acid (ABA), brassinosteroid (BR), ethylene (ET), and cytokinin (CK). In cotton, the functional peptide GhPSK promotes fibre growth, likely through the stimulation of auxin and BR signalling pathways (Han et al. 2014). Another example is the small peptide AtZSP1, which promotes organ growth, likely through modulation of cytokinin levels in shoots (Zeng et al. 2022). Additionally, the 36‐amino acid peptide PLS, encoded by a lncRNA, regulates both root and leaf development by repressing ET responses (Casson et al. 2002). These findings underscore the integration of peptide signalling with hormone pathways and temporal regulation, revealing practical targets for crop improvement.

### 4.2 Roles in Reproduction and Fertilisation

Plant reproduction is tightly regulated by small peptides that coordinate male–female interactions, pollen tube guidance, gamete delivery, and early embryo development (Figure 3). These peptides act as crucial signalling intermediates between reproductive organs and developing tissues, ensuring species specificity, successful fertilisation, and viable seed formation (Kim, Jeon, Oh, et al. 2021; Takeuchi 2021; Zhang, Yue, et al. 2021). One striking example is the interaction between POLLEN COAT PROTEIN‐B (PCP‐B) peptides and the stigma‐derived peptide RALF33. PCP‐B competes for binding to the ANJ/FER receptor complex, thereby suppressing reactive oxygen species (ROS) production in papillary cells and facilitating pollen hydration (Liu, Shen, et al. 2021). This finely tuned mechanism illustrates how reproductive success is tightly controlled by competitive receptor‐ligand dynamics. In Brassicaceae, the SCR/SP11, expressed on the pollen surface, binds to SRK receptor kinases on the stigma, thereby initiating a self‐incompatibility response that prevents inbreeding (Takayama et al. 2001).

During the later stages of fertilisation, RALF peptides play essential roles in maintaining pollen tube integrity and ensuring precise delivery of sperm cells (Covey et al. 2010; Chevalier et al. 2013; Kou et al. 2021). They function through receptor complexes involving ANX1‐BUPS2 and FER‐ANJ‐HERK1, safeguarding tube growth and coordinating the final steps of fertilisation (Ge et al. 2017; Zhong et al. 2022). To ensure reproductive isolation and species‐specific fertilisation, the defensin‐like attractant peptide AtLURE1 is secreted by synergid cells in Arabidopsis. It acts as a chemoattractant, guiding pollen tubes from the same species and thereby maintaining mating fidelity (Liu, Wang, et al. 2021).

Following fertilisation, peptide signals continue to guide embryonic development. CLE8 and CLE19 are two such peptides that contribute to seed size determination and proper patterning of embryonic structures. CLE8 expression in early embryos promotes *WOX8* transcription, which governs cell fate decisions during embryo development (Fiume and Fletcher 2012). Furthermore, recent studies have revealed several instances of peptide–hormone crosstalk during plant reproduction and fertilisation. For example, CLE19 peptide signalling suppresses BR output to maintain pollen exine homeostasis (Wang et al. 2025). In addition to these CPs, a remarkable case of NCPs is the microRPG1 peptide, a 31‐amino acid NCP unique to the *Zea genus*. It modulates kernel dehydration rate, a crucial post‐harvest trait, by influencing ET signalling and is functionally conserved even in Arabidopsis, suggesting broader utility in crop improvement (Yu et al. 2025). These findings provide mechanistic insight into how peptide–hormone signals are integrated in plant reproduction.

### 4.3 Mediation of Abiotic Stress Responses

**FIGURE 4:**
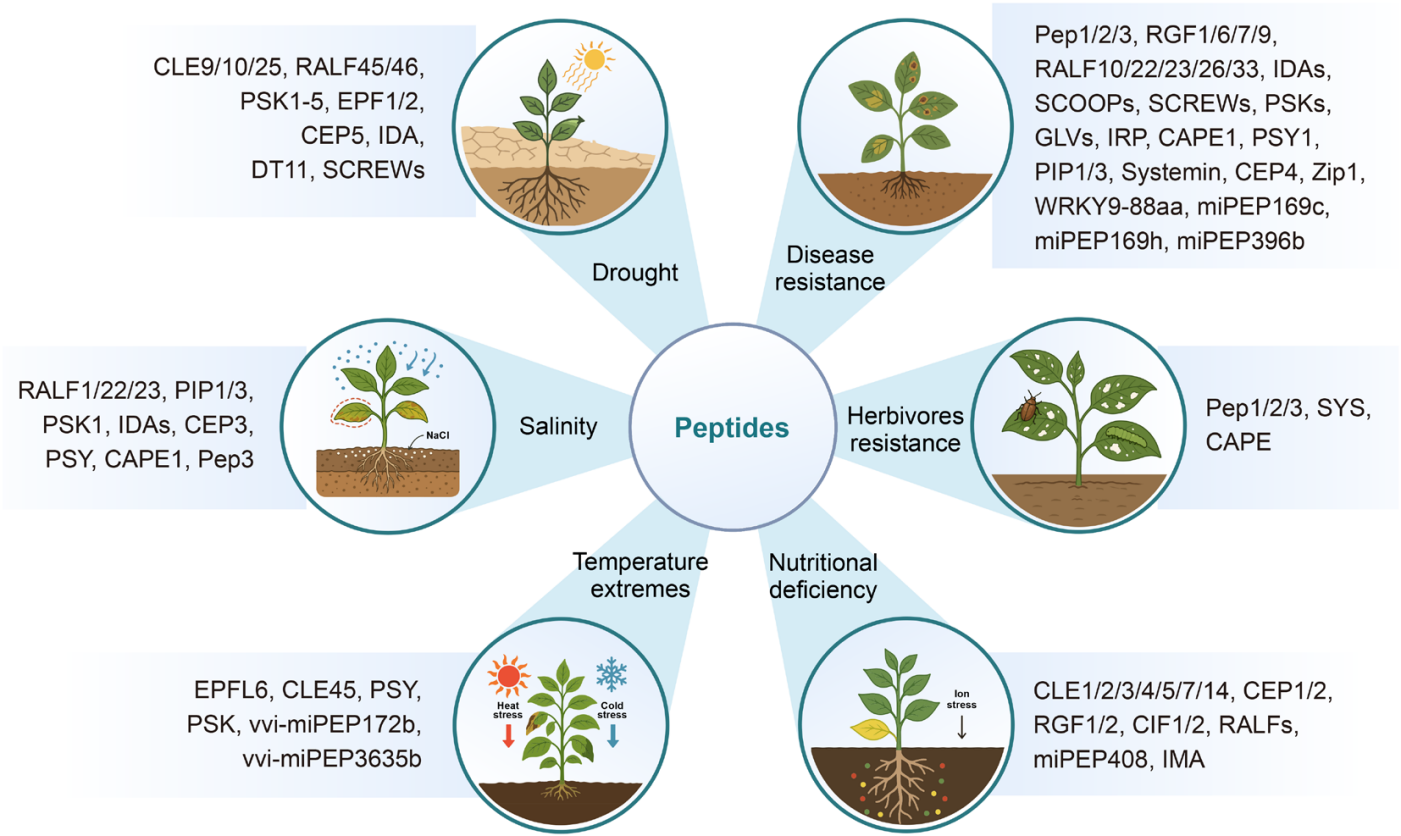
Plant peptides regulate both abiotic and biotic stress responses. The schematic diagram categorises representative plant peptides based on their roles in abiotic and biotic response. Peptides mediate responses to drought, salinity, temperature extremes, nutrient deficiency, disease, and herbivore resistance. The recurrence of peptide families like PSK, RALFs, and CEPs across multiple contexts underscores their functional versatility in plants.

Plants frequently encounter environmental stresses such as drought, salinity, and temperature extremes, which significantly impact growth and productivity. Small peptides have emerged as pivotal regulators in these abiotic stress responses (Figure 4), integrating external cues with hormonal signalling networks (Jones et al. 2021; Zhang, Han, et al. 2025). CLE45, a pistil‐specific peptide, plays a protective role under heat stress by maintaining pollen tube viability, thus safeguarding reproductive success at elevated temperatures (Endo et al. 2013). Bna.EPF2, a homologue of EPF peptides, enhances water use efficiency and drought tolerance by regulating stomatal density and size without compromising yield in *Brassica napus* (Jiao et al. 2023). In addition, CEPs, Peps, IDAs, PSYs, and RALF peptides have been shown to regulate salt and drought tolerance, likely by modulating ion transport and osmotic homeostasis pathways (Feng et al. 2018; Nakaminami et al. 2018; Zhao et al. 2018; Guo et al. 2021; Stührwohldt et al. 2021; Liu, Shen, et al. 2022; Shen, Zuo, et al. 2023). Beyond CPs, NCPs are increasingly recognised as novel abiotic stress regulators. For instance, vvi‐miPEP172b and vvi‐miPEP3635b, derived from the primary transcript of miR72b and miR3635b respectively, enhance cold tolerance by modulating oxidative stress‐related genes and boosting antioxidant capacity (Chen et al. 2022). A large‐scale peptidomic study further identified over 500 stress‐responsive peptides in Arabidopsis, many of which originate from non‐coding or intergenic regions and are predicted to function as regulatory NCPs (De Coninck et al. 2013). These peptides signalling exemplifies how plants adapt their physiology through systemic cues.

Recently, studies have shown that certain peptides contribute to drought stress by altering hormone accumulation in plants. For example, upon drought exposure, CLE25 is expressed in the roots and translocated via the vascular system to the leaves, where it promotes ABA biosynthesis by upregulating *NCED3*, leading to stomatal closure and enhanced drought tolerance (Takahashi et al. 2018; Zhang, Shi, et al. 2019). In rice, a gene *OsDT11* encoding a cysteine‐rich peptide was identified to be involved in regulating drought tolerance. Overexpression of *OsDT11* significantly increased ABA content and markedly enhanced drought tolerance (Li et al. 2017). Moreover, CEP5 signalling is relevant for osmotic and drought stress tolerance by counteracting the impacts of auxin in Arabidopsis (Chapman et al. 2020).

### 4.4 Coordination of Biotic Stress Responses

In the face of pathogenic attacks, plants deploy a robust innate immune system that involves recognition of both exogenous (PAMPs) and endogenous (DAMPs) signals (Khan, Islam, et al. 2022; Wang, Cang, et al. 2024). Within this system, endogenous peptides, particularly those induced by stress or pathogen exposure, serve as immune amplifiers or modulators within these pathways (Hu, Zhang, and Shi 2018; Hou, Liu, and He 2021; Rzemieniewski and Stegmann 2022; del Corpo et al. 2024) (Figure 4). Immunomodulatory peptides can be broadly categorised by the presence or absence of signal peptides. Signal peptide‐containing families such as PSKs, PAMP‐induced secreted peptides (PIPs), PSYs, SCOOPs, GLVs, RALFs, SMALL PHYTOCYTOKINES REGULATING DEFENCE AND WATER LOSS (SCREWs), and immune response peptides (IRPs) are typically secreted into the apoplast and perceived by plasma membrane‐localised receptor‐like kinases (RLKs) (Igarashi et al. 2012; Hou et al. 2014; Stegmann et al. 2017; Wang, Yao, et al. 2020; Hou, Liu, Huang, et al. 2021; Rhodes et al. 2021; Liu, Hou, et al. 2022; Rzemieniewski and Stegmann 2022; Stegmann et al. 2022). These peptides reinforce immune activation, often by priming downstream signalling components like mitogen‐activated protein kinases (MAPKs), calcium channels, and the production of ROS and plant hormones. Exogenous application of the PSK peptide boosts tomato resistance against *Botrytis cinerea*, whereas downregulation of PSK gene expression heightens plant susceptibility to the pathogen (Zhang et al. 2018). In addition, signal peptide‐lacking peptides such as systemin, Peps, and Zip1 are secreted via unconventional pathways and function as DAMPs (Huffaker et al. 2006; Ziemann et al. 2018; Zhang, Zhang, and Lin 2020).

The brown planthopper ( *Nilaparvata lugens* ) is one of the most destructive sap‐sucking pests threatening rice production. *N. lugens* can induce the expression of multiple PEP precursor genes and PEP receptor genes in rice leaf sheaths. CRISPR/Cas9‐mediated knockout of rice OsPEPR1/OsPEPR2 genes significantly weakens rice resistance to brown planthopper, whereas exogenous application of the synthetic small peptide OsPep3, which exhibits strong elicitor activity, markedly enhances rice resistance against this pest (Shen et al. 2022). Furthermore, cyclotides act as host‐defence agents against insects by interacting with cellular membranes and interfering with their normal biological activities (Pelegrini et al. 2007; Huang et al. 2019). For instance, Vcom1 and Vcom2 from *Viola communis* showed cytotoxicity to Sf9 insect cell lines, suggesting that they could be further explored as insecticidal agents (Khatibi et al. 2025).

Increasing evidence suggests that NCPs are functionally relevant in biotic defence. For instance, PSK‐α, encoded by lncRNA7, has been shown to alleviate fungal disease symptoms in cotton by modulating defence hormone pathways (Zhang et al. 2022). Additionally, a high‐throughput screen of 89 Arabidopsis miPEPs identified 17 functional candidates, among which miPEP169c, miPEP169h, and miPEP396b significantly upregulated *PR1*, a key pathogenesis‐related gene, thereby enhancing disease resistance (Ormancey et al. 2024). These discoveries underscore the regulatory potential of NCPs in fine‐tuning immune outputs and offer new entry points for crop disease resistance engineering.

### 4.5 Antimicrobial Peptides (AMPs)

**FIGURE 5:**
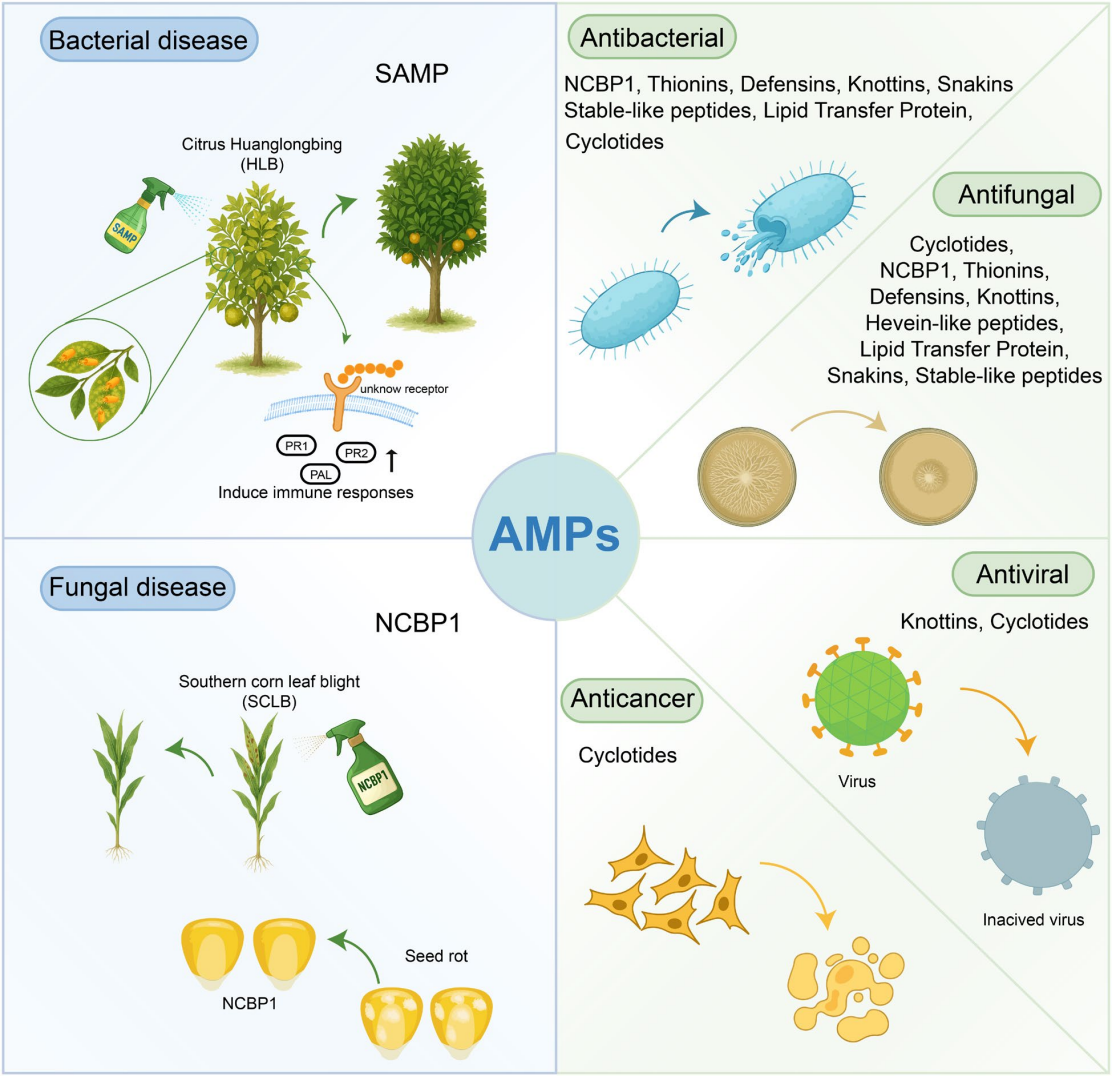
The dual function of plant‐derived antimicrobial peptides (AMPs). Plant‐derived antimicrobial peptides (AMPs) exhibit dual functionality. Their primary role involves direct microbial inhibition or killing, predominantly through disruption of microbial membranes. Beyond this direct antimicrobial activity, AMPs serve as critical modulators of broader plant immune responses against bacterial and fungal pathogens. This integrated defence strategy establishes AMPs as multifunctional molecules essential for plant resistance.

Plant‐derived AMPs are a fundamental component of the plant innate immune system, representing a diverse group of small molecules that protect against a broad‐spectrum of pathogens including bacteria, fungi, oomycetes, and virus (Moghaddam et al. 2016; Iralu et al. 2024; Sun et al. 2025). Their primary mode of action involves direct microbial inhibition or killing, most commonly through disruption of the microbial membrane (Li, Zuo, et al. 2022; Nayab et al. 2022) (Figure 5). Cationic AMPs, such as defensins and thionins, interact electrostatically with negatively charged components of microbial membranes, such as phospholipids, lipopolysaccharides, and teichoic acids. This interaction facilitates their insertion into membranes, resulting in pore formation via barrel‐stave, toroidal‐pore, or carpet‐like models, membrane thinning, or micellization, ultimately causing leakage of cellular contents and cell death (Iqbal et al. 2019; Khan et al. 2019). Although traditionally regarded as direct microbicidal agents, AMPs are now recognised as multifunctional molecules that also serve as key modulators of plant immune response (Campos et al. 2018) (Figure 5). For instance, stable antimicrobial peptides (SAMPs) from *Microcitrus australasica* not only reduce *Liberibacter crescens* titers and disease symptoms in Citrus Huanglongbing (HLB)‐infected trees but also trigger innate immune responses that inhibit further infections (Huang, Araujo, et al. 2021; Wang et al. 2021).

Plant‐derived AMPs are classified into several families based on their sequence and structural characteristics, including thionins, defensins, hevein‐like peptides, knottins, stable‐like peptides, snakins, and cyclotides (Büyükkiraz and Kesmen 2022). Thionins, among the most prominent plant AMPs, are rich in cysteine, lysine, and arginine residues and exhibit strong antibacterial and antifungal activities (Nawrot et al. 2014; Höng et al. 2021). Defensins and hevein‐like peptides play critical roles by disrupting microbial membranes (Gao et al. 2000; Shukurov et al. 2012; Sagehashi et al. 2017; Velivelli et al. 2020; Alhhazmi et al. 2024). Knottins display cytotoxic and antimicrobial properties (Li, Hu, et al. 2021; Attah et al. 2022). Stable‐like peptides, characterised by a conserved cysteine motif, represent a heterogeneous group with broad biological activities, including antifungal, antibacterial, trypsin‐inhibitory, and ribosome‐inactivating functions (Slavokhotova and Rogozhin 2020; Lima et al. 2022). Snakins are distinguished by the highest number of cysteine residues and disulfide bonds among plant AMPs, which confer structural stability and are closely linked to their antimicrobial functions (Oliveira‐Lima et al. 2017; Su et al. 2020). Cyclotides exert antimicrobial effects by disrupting microbial membranes through pore formation and destabilisation (Pelegrini et al. 2007; Slazak et al. 2016; Weidmann and Craik 2016; Pinto et al. 2018).

Beyond CPs, recent studies have revealed that NCPs can also show antimicrobial activity. Our previous work uncovered 25 NCPs with broad‐spectrum antifungal activity, varying in efficacy across different fungal species (Tian et al. 2021). Among them, Non‐Canonical Antibacterial Peptide 1 (NCBP1) demonstrated potent antibacterial activity against both Gram‐positive and Gram‐negative bacteria by targeting bacterial membrane lipids, specifically phosphatidylglycerol and cardiolipin. NCBP1 not only exhibited therapeutic efficacy in murine models but also effectively suppressed fungal pathogen growth and enhanced disease resistance in maize (Chen et al. 2025). AMPs represent a promising alternative to conventional antibiotics, primarily due to their remarkably low propensity for inducing microbial resistance and their efficacy against multidrug‐resistant pathogens. These results highlight the applied potential of AMPs in crops and biopharmaceuticals.

It was noting that CPs primarily act as signalling ligands that bind to RLKs (Olsson et al. 2019; Lalun and Butenko 2025). Ligand binding typically induces heterodimerization with co‐receptors such as somatic embryogenesis receptor kinases (SERKs), initiating intracellular signal relay through phosphorylation cascades (He et al. 2018). This transduction involves receptor‐like cytoplasmic kinases (RLCKs), which activate shared downstream nodes including MAPKs and calcium‐dependent protein kinases (CDPKs) (Lin et al. 2013). These kinases phosphorylate key transcription factors (e.g., WRKYs) to regulate diverse developmental and immune outputs (Tabata et al. 2014; He et al. 2018; Khan, Zhang, et al. 2022; Selby and Jones 2023; Li, Li, et al. 2024; Lin et al. 2024). Notably, peptide–receptor relationships are not strictly one‐to‐one; multiple ligands often converge on common co‐receptors and signalling modules. Specificity in cellular responses is achieved through contextual mechanisms such as spatiotemporal expression, competitive receptor binding, selective co‐receptor recruitment, and the engagement of distinct downstream components (He et al. 2018; Olsson et al. 2019).

## 5 Application of Plant Peptides

Plant‐derived peptides have emerged as multifunctional agents with broad applications in agriculture and biomedicine. Beyond their fundamental roles in regulating development and stress responses, these small molecules offer practical solutions for improving crop performance and human health. This section outlines the current and potential applications of plant peptides, focusing on elite genetic variation, genetic engineering of peptides, exogenous treatment strategies, and pharmaceutical uses.

### 5.1 Exploiting Natural Variation for Crop Improvement

**FIGURE 6:**
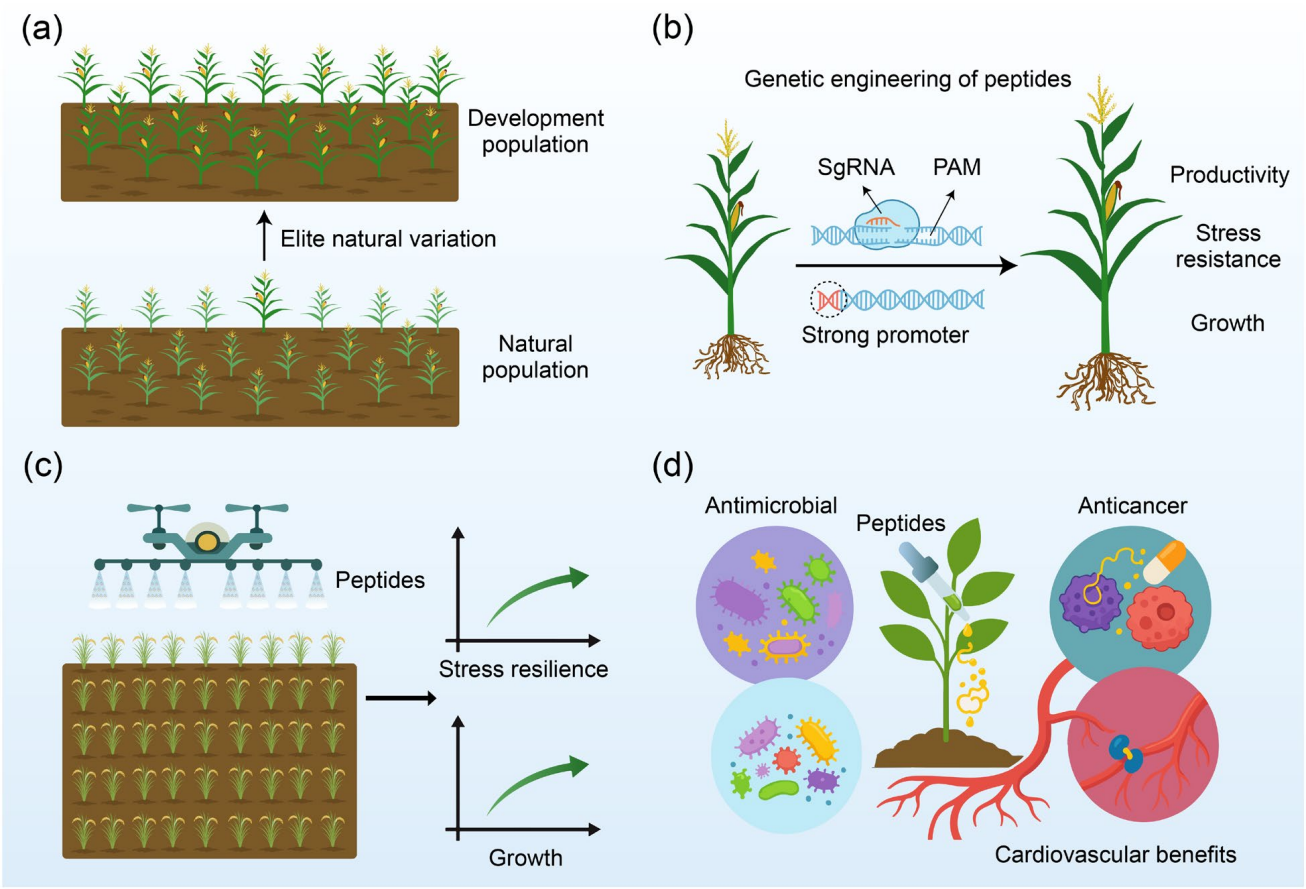
Application strategies of plant peptides in agriculture and biomedicine. (a) Natural variation in peptide‐coding regions contributes to trait diversity, including nutrient efficiency and stress resilience. Mining elite alleles facilitates crop improvement through molecular breeding. (b) Genetic engineering of peptide‐coding sequences or regulatory elements enables precise manipulation of growth, stress resistance, and productivity. (c) Exogenous application of synthetic or natural peptides via foliar spray or seed treatment offers a non‐transgenic route to boost plant growth and resistance. (d) In biomedicine, plant‐derived peptides exhibit a broad spectrum of bioactivities, including antimicrobial, anticancer, and cardiovascular benefits, with emerging potential as therapeutic agents and functional food components.

Harnessing naturally occurring peptide diversity is a promising strategy for crop trait enhancement. Variations in peptide sequence, length, translational efficiency, and upstream regulatory elements can significantly impact plant phenotypes. Recent studies have demonstrated that elite alleles of peptide‐encoding loci are linked to key agronomic traits (Figure 6a). For instance, a single nucleotide polymorphism in the uORF of *GmPHF1* modulates phosphate acquisition efficiency in soybean, offering a path toward sustainable nutrient management (Guo et al. 2022). Similarly, natural variation in the microRPG1 peptide, involved in kernel dehydration rate in maize, reveals how de novo evolved NCPs can be harnessed to optimise post‐harvest traits (Yu et al. 2025). These findings emphasise the need to systematically screen peptide diversity in natural populations and landraces, which may harbour valuable alleles for breeding climate‐resilient and high‐yield crops.

### 5.2 Genetic Engineering of Peptides

The advent of CRISPR‐mediated genome editing has enabled precise manipulation of peptide genes, including their regulatory elements and coding sequences (Zhang, Zhang, et al. 2021; Neelakandan et al. 2022). This has opened new avenues for targeted trait engineering with minimal off‐target effects (Figure 6b). For example, CRISPR‐mediated knockout of OsEPFL6, OsEPFL7, and OsEPFL9 generated rice triple mutants with improved panicle architecture and higher yield, highlighting the value of targeting peptide genes for developmental reprogramming (Guo, Lu, et al. 2023). CRISPR technology also offers the potential to edit peptide uORFs or sORFs, influencing translational control or generating novel bioactive peptides. This precision allows breeders to uncouple yield and defence traits, thereby optimising plant performance under diverse environmental conditions.

In addition to CRISPR‐mediated genome editing, the transgenic expression of peptides has emerged as a promising strategy for enhancing disease resistance in plants (Figure 6b). A notable example is the *ProTBF1‐uORFsTBF1* regulatory cassette from Arabidopsis, which integrates transcriptional and translational control to fine‐tune the expression of immune regulators. When introduced into rice and Arabidopsis, this cassette conferred robust disease resistance without compromising plant growth by tightly regulating genes such as *SNC1* and *NPR1* (Xu, Greene, et al. 2017; Xu, Uan, et al. 2017). Additionally, the expression of the plant defensin *NaD1* in cotton conferred resistance to *Fusarium oxysporum* and *Verticillium dahlia* (Gaspar et al. 2014). Similarly, transgenic rice expressing the AMP *snakin‐1* exhibited significant disease resistance with no adverse effects on yield or other agronomic traits (Das et al. 2021). Collectively, these findings underscore the potential of engineered peptide expression as a sustainable and effective alternative to chemical pesticides for plant disease management.

### 5.3 Exogenous Peptide Applications in Crop Management

In addition to genetic manipulation, exogenous application of peptides represents a non‐transgenic, rapid‐response strategy to enhance plant health and productivity (Figure 6c). Peptides can be delivered via foliar sprays, seed priming, or soil amendments to influence growth, immunity, and stress resilience. Growth‐regulating peptides, such as PSKs and CEPs, have been shown to stimulate root development and nutrient uptake when applied exogenously (Lu and Xiao 2024). Likewise, owing to their stability, low toxicity, and high specificity, peptides are attractive candidates for commercial formulation and field deployment. For instance, precision trunk injection of AMPs is being explored to treat citrus trees affected by HLB (Huang, Araujo, et al. 2021; Ojo et al. 2024). Cyclotides are particularly promising for science and agriculture, serving as a class of defensive peptides that naturally protect plants against pests. Companies like Vestaron and Innovate Ag have successfully developed various peptide‐based insecticides derived from both animal and plant sources. These novel agents provide effective pest control while maintaining safety and sustainability for pollinators, beneficial organisms, and local biodiversity. They thus represent a critical tool in meeting the challenge of environmentally friendly and sustainable food security for a growing global population. Moreover, peptide application can prime systemic resistance responses or modulate hormonal balance under abiotic stress conditions, providing a rapid physiological adjustment to adverse environments.

### 5.4 Biomedical Potential of Plant‐Derived Peptides

Beyond plant systems, plant peptides also hold transformative potential in pharmaceutical and nutraceutical development (Figure 6d). These bioactive molecules display diverse properties, including antimicrobial, anticancer, antihypertensive, and cholesterol‐lowering activities. For example, the non‐canonical antibacterial peptide NCBP1 demonstrates broad‐spectrum efficacy against Gram‐positive and Gram‐negative bacteria and shows therapeutic promise in murine models of multidrug‐resistant infections (Chen et al. 2025). In clinical contexts, specific peptides from soy, flaxseed, and other edible plants have been shown to modulate key physiological targets such as angiotensin‐converting enzyme and renin, supporting cardiovascular health. Functional food products enriched with such peptides are gaining traction as dietary interventions for hypertension, diabetes, and metabolic syndrome (Iglesias et al. 2025). Cyclotides, with their unique cyclic cystine knot structure, have established themselves as a premier scaffold for epitope grafting in peptide drug design (Gerlach et al. 2010). Their exceptional stability, proteolytic resistance, and tolerance for sequence insertion into various loops enable the stabilisation of diverse bioactive epitopes against degradation (Poth et al. 2013; Craik and Du 2017). The recent development of modular strategies, such as the “plug and play” approach, allows for the efficient synthesis of previously inaccessible peptides with nanomolar affinities (Koehbach et al. 2024). These advances are therefore unlocking the full potential of cyclotides as stabilised, potent therapeutics against diverse targets, thereby accelerating their translation into clinical applications.

## 6 Challenges and Future Perspectives

Plant peptide research offers significant potential for advancing agriculture and biomedicine. While substantial progress has been made in identifying peptides and characterising their extracellular signalling mechanisms, key challenges persist. First, the intracellular functions of specific peptides remain incompletely understood. Although most plant peptides act via membrane‐bound receptors in the apoplast, emerging evidence suggests a subset, particularly NCPs lacking N‐terminal signal peptides, may function within the cytoplasm (Ren et al. 2021). However, the molecular mechanisms underlying such intracellular activities are largely unexplored and often speculative due to limited direct evidence. Future research must prioritise elucidating the subcellular localization and potential non‐canonical roles of plant peptides using advanced imaging, interactome mapping, and synthetic biology. Integrative platforms combining multiple‐omics and machine learning are also essential to predict and verify intracellular peptide functions. Understanding this layer of peptide‐mediated regulation could unlock new strategies for enhancing plant resilience and metabolic precision.

A second major challenge involves deciphering the specific functions of individual peptides within families containing numerous paralogs exhibiting functional redundancy or overlap (Xiao et al. 2025). Furthermore, establishing the physiological relevance of NCPs encoded by sORFs in UTRs, lncRNAs, circRNAs, or pri‐miRNAs is particularly difficult. This requires rigorous genetic and biochemical evidence beyond mere detection to demonstrate endogenous production at functional levels, define their mechanisms of action (often distinct from CPs), and definitively link them to phenotypes. Gene‐editing technologies, especially the expanding CRISPR‐Cas toolbox, provide powerful methods to dissect peptide function. Precise genomic modifications, including knockouts, knock‐ins, and regulatory region editing, enable functional analysis of peptide‐encoding genes and their receptors, facilitating the dissection of redundancy and complex signalling networks in plants.

Moreover, the application of peptides in agriculture faces several key challenges, including limited peptide stability, inefficient delivery across plant tissues, and the high cost of large‐scale production (Zhang, Han, et al. 2025). The large‐scale production of complex plant peptides, particularly those requiring specific PTMs for bioactivity, remains economically unfeasible (Chaudhary et al. 2024). Translating plant‐derived antimicrobial or therapeutic peptides into clinical applications also entails navigating stringent regulatory requirements related to efficacy, safety, pharmacokinetics, and scalable manufacturing. A significant advantage of peptides, compared to those exerting direct toxic effects, lies in their mode of action. Peptides typically function through high‐affinity, specific interactions and can elicit potent biological responses at minimal doses. This efficiency offers a strategic advantage, making peptides an attractive modality in agricultural biotechnology and drug discovery, where achieving desired activity with lower product volumes aligns with economic and manufacturing realities. Looking ahead, artificial intelligence (AI) and machine learning are poised to revolutionise the field by modelling peptide–receptor interactions and signalling pathways through the integration of multi‐omics data. AI‐driven tools will facilitate the rational design of peptides with improved stability, receptor specificity, and bioactivity, as well as optimise CRISPR target selection for crop improvement. Future efforts may also focus on enhancing peptide stability and delivery. Recent breakthroughs in prokaryotic and eukaryotic expression systems, combined with advanced delivery systems, will be essential for effective application in both agricultural and biomedical settings.

While challenges remain in understanding peptide biology and translating discoveries into practical applications, the convergence of gene editing, AI, advanced sequencing, and bioengineering provides an unprecedented toolkit. By strategically integrating these technologies, plant peptide research is poised to drive innovation in sustainable agriculture and next‐generation therapeutics. The future lies in intelligent integration, precise manipulation, and translational application of plant peptide systems.

## Author Contributions

L.W., S.W., J.Z., and M.C. planned the review outline. S.W., J.Z., M.C., B.Z., H.Z. and L.W. wrote the majority of the manuscript and prepared the figures. S.W., J.Z., M.C., B.Z. and L.W. prepared Tables. L.W., S.W., M.C. and J.Z. revised the manuscript. All authors read and approved the final manuscript.

## Funding

This work was supported by National Natural Science Foundation of China, U22A20474, 32172073. National Key Research and Development Program of China, 2022YFD1201802. Key Scientific and Technological Project of Henan Province Department of China, 242102111129.

## Conflicts of Interest

The authors declare no conflicts of interest.

## Data Availability

The data that support the findings of this study are available on request from the corresponding author. The data are not publicly available due to privacy or ethical restrictions.
